# Gamma-glutamyl transferase levels are associated with the occurrence of post-stroke cognitive impairment: a multicenter cohort study

**DOI:** 10.1186/s12883-022-02587-4

**Published:** 2022-02-23

**Authors:** Siqi Li, Xiaoling Liao, Yuesong Pan, Xianglong Xiang, Yumei Zhang

**Affiliations:** 1 Department of Neurology, Beijing Tiantan Hospital, Capital Medical University, Beijing, China; 2 China National Clinical Research Center for Neurological Diseases, Capital Medical University, Beijing, China; 3 Department of Rehabilitation Medicine, Beijing Tiantan Hospital, Capital Medical University, Beijing, China

**Keywords:** Association, Gamma-glutamyl transferase, Post-stroke cognitive impairment

## Abstract

**Background:**

Gamma-glutamyl transferase (GGT) is involved in maintenance of physiological concentrations of glutathione in cells, and protects them from oxidative stress-induced damage. However, its role in post-stroke cognitive impairment (PSCI) remains unknown. Here, we investigated the effects of serum GGT on PSCI.

**Methods:**

We conducted a prospective, multicenter cohort study. A total of 1, 957 participants with a minor ischemic stroke or transient ischemic attack whose baseline GGT levels were measured were enrolled from the Impairment of Cognition and Sleep (ICONS) study of the China National Stroke Registry-3 (CNSR-3). They were categorized into four groups according to quartiles of baseline GGT levels. Cognitive functions were assessed using the Montreal Cognitive Assessment (MoCA) approach. Multiple logistic regression models were performed to evaluate the relationship between GGT and PSCI at 3 months follow-up.

**Results:**

Among the 1957 participants, 671 (34.29%) patients suffered PSCI at 3 months follow-up. The highest GGT level quartile group exhibited a lower risk of PSCI in the fully adjusted model [OR (95% CI): 0.69 (0.50-0.96)], relative to the lowest group. Moreover, incorporation of GGT to the conventional model resulted in slight improvements in PSCI outcomes after 3 months (NRI: 12.00%; IDI: 0.30%).

**Conclusions:**

Serum GGT levels are inversely associated with the risk of PSCI, with extremely low levels being viable risk factors for PSCI.

**Supplementary Information:**

The online version contains supplementary material available at 10.1186/s12883-022-02587-4.

## Background

Globally, stroke is a leading cause of disabilities and mortalities, affecting one in every four people [1–3]. Cognitive impairment, which is a common stroke complication, has attracted numerous research attention. According to the Vascular Impairment of Cognition Classification Consensus Study (VICCCS), vascular cognitive impairment (VCI) refers to cognitive disorders caused by underlying vascular factors and can be associated with obvious cerebrovascular diseases [4]. Vascular cognitive impairment no-dementia (VCI-ND) is based on the proposed criteria of small vessel ischemic disease and cognitive deficits without dementia [5]. Furthermore, vascular depression can lead to decreased intracortical facilitation and disruption of glutamate neurotransmission, which plays a major role in synaptic plasticity, and might contribute to the cognitive deterioration [6–8]. These cognitive and mood symptoms are associated with vascular damage in the white matter connecting the prefrontal cortex and basal ganglia as well as those connecting the prefrontal cortex and cerebellum. Transcranial doppler ultrasound revealed a hemodynamic pattern of cerebral hypoperfusion and increased vascular resistance [9]. Cognitive impairment manifests as memory decline, abstract thinking, and judgment impairment, but, the ability for daily life is normal. However, it presents a higher risk for more severe cognitive impairments, especially after recurrent strokes, which can seriously affect a patient’s quality of life. As a subtype of VCI, post-stroke cognitive impairment (PSCI) emphasizes that stroke events trigger cognitive dysfunction. Approximately 50% of stroke survivors manifest cognitive dysfunctions, 6 months after stroke, and are more likely to develop dementia within the following 3 years, which significantly affects their quality of life [10, 11]. Moreover, a community-based epidemiological survey in China reported that incidences of PSCI and dementia were 56.6 and 23.2%, respectively, 3 months after stroke [12].

Currently, the diagnosis of PSCI is mainly based on clinical manifestations and on structural changes in brains of patients. This diagnostic criteria formed the basis for construction of SIGNAL_2_ and CHANGE risk models [13, 14]. The Leukoaraiosis and Disability Study (LADIS) revealed that the severity of changes in white matter is associated with worse performances on overall cognitive tests [15]. Alterrations in mean diffusivity of normal-appearing white matter, corpus callosum atrophy, the presence of lacunes in the thalamus, gray matter, and hippocampal volumes are significantly associated with speed, memory performance, and executive functions. Combined measurement of these imaging metrics can be used as a comprehensive neuroimaging marker for predicting vascular cognitive impairment [16–19]. However, the use of biomarkers for the diagnosis and prognosis of PSCI remains a challenge [20, 21].

Gamma-glutamyl transferase (GGT) is a serum metabolic biomarker that is mainly used to assess liver function [22, 23]. GGT is involved in maintenance of physiological concentrations of glutathione in cells and reflects the oxidation-antioxidant balance in the body [24, 25]. It has been reported that GGT levels are correlated with decreased cognitive function in diabetics [26, 27]. In addition, a Korean retrospective study found that GGT variability is associated with Alzheimer’s disease, implying that serum GGT levels are potential predictors of cognitive decline [28]. Moreover, serum metabolites, including GGT, have been shown to be differentially expressed in patients with PSCI and post-stroke non-cognitive impairment [29, 30], suggesting that GGT may affect PSCI occurrence.

However, the role of GGT in PSCI has not been conclusively determined, and to date, only a handful of models for predicting PSCI have been constructed. Notably, these models are mainly constructed based on cerebrovascular risk factors, with the effects of non-cerebrovascular risk factors on PSCI remaining unclear. Therefore, the relationship between GGT and PSCI should be evaluated further. In addition, expert consensus states that the diagnosis of PSCI refers to cognitive dysfunction after a stroke event in 6 months, and most patients suffer cognitive impairment within 3 months after stroke [4–6]. Therefore, we aimed to investigate the association of serum GGT with PSCI during 3 months of follow-up. This study is presented in accordance with the STROBE reporting checklist.

## Methods

### Study population

All participants with a minor ischemic stroke or transient ischemic attack were selected from the Impairment of Cognition and Sleep (ICONS) study of the China National Stroke Registry-3 (CNSR-3). Patient selection was performed from 2015 to 2018 [31]. ICONS is a large national, multi-center, and prospective cohort involving about 40 hospitals in China [32]. Acute ischemic stroke (AIS) and transient ischemic attack (TIA) are the most common cerebrovascular events in China. PSCI includes cognitive impairment caused by AIS and TIA. Studies report that 3 months after TIA, more than one-third of patients exhibit cognitive dysfunction [33–35]. Therefore, we continuously recruited patients with AIS and TIA, with no history of cognitive disorders before stroke. Generally, according to the World Health Organization criteria, AIS and TIA are diagnosed based on symptomatic presentations (acute onset of neurological deficits, which persist for > 24 h in the case of AIS, or for < 24 h in the case of TIA), physical signs, scale evaluations, and are confirmed by neuroimages (magnetic resonance or brain computed tomography) [36–38]. 

The inclusion criteria for patients in this study were: (i) Diagnosed with AIS or TIA and hospitalized upon symptomatic onset within 7 days; (ii) The absence of any history of cognitive dysfunctions, serious mental disorders such as psychosis or schizophrenia (documented in medical records); (iii) The absence of any other factors that affect cognitive or sleep assessments, for instance, severe aphasia defined as National Institutes of Health Stroke Scale (NIHSS) item 9 (Best Language) > 2, consciousness disorders defined as NIHSS item 1a (Level of Consciousness) > 1 or 1b (LOC Questions) > 1, hearing loss, visual impairment, hard to cooperate, severe unilateral neglect or dyslexia; (iv) Muscle strength of handedness ≥ level 4 after Manual Muscle Testing; and (v) Those whose baseline GGT levels were accessible and who had completed the standard cognitive function evaluation at 3 months of follow-up. Eventually, a total of 1957 participants were enrolled in our study.

This study was performed in accordance with the guidelines described by the Helsinki Declaration and was approved by the Ethical Committee of Beijing Tiantan Hospital (No. KY2015-001-01). Prior to their inclusion in the study, all participants signed written informed consents.

### Data collection

The data collection protocol and statistical analyses were performed as previously described [36, 37]. Confounding variables in this study were selected based on the findings of studies on risk factors for PSCI [12, 39–43]. Upon admission, all participants were comprehensively and precisely assessed, which included the collection of their demographic information (age, sex, body mass index, smoking, and educational level, among others), and evaluation of their medical histories (stroke, hypertension, dyslipidemia, diabetes mellitus, coronary artery disease, atrial fibrillation, heart failure, fatty liver disease, epilepsy, and cancer). The 7-item Generalized Anxiety Disorder Scale was used to assess participants’ anxiety status. In addition, they were subjected to a detailed physical examination, and several parameters, including the modified Rankin Scale, Trial of ORG 10172 in Acute Stroke Treatment (TOAST) type, NIHSS score, ABCD2score, Glasgow Coma Scale, and Manual Muscle Testing were assessed. Moreover, exposure to various medications during hospitalization (antiplatelet aggregation therapy, antihypertensive therapy, lipid-lowering therapy, hypoglycemic therapy, antidepressant therapy, sedative-hypnotic therapy) was assessed. Then, fasting blood samples were obtained, for laboratory analysis of serum GGT, high-density lipoprotein (HDL), low-density lipoprotein (LDL), triglycerides (TG), total cholesterol (TC), alanine aminotransferase (ALT), aspartate aminotransferase (AST), serum albumin, effective glomerular filtration rate (eGFR), albumin, and serum uric acid (UA) levels. These samples were collected in EDTA anticoagulation blood collection and serum-separation tubes within 24 h of admission.

### Outcome evaluation

Clinical outcome has involved the assessment of PSCI occurrence after 3 months of follow-up. We applied the Montreal Cognitive Assessment (MoCA) approach to assess cognitive functions and adopted a MoCA cut-off point of <23/30, which has previously been shown to have the best sensitivity and specificity for detecting PSCI in Chinese patients [12, 35, 44–46]. Baseline MOCA evaluation was performed by a certified neuropsychologist, while follow-up MoCA evaluation was performed by a neurologist who was blinded to baseline assessment.

### Statistical analyses

All statistical analyses were conducted using SAS version 9.4 (SAS Institute Inc., Cary, NC, USA). Participants who were lost to follow-up were excluded from the study. Continuous variables are presented as median (interquartile range) and were compared using the Kruskal–Wallis test. Categorical variables are expressed as numbers (proportions) and were compared using the χ2 or Fisher’s exact tests. First, we categorized all recruited participants into four groups according to baseline GGT quartiles, then, we collected their characteristics upon admission. Thereafter, we analyzed the association between GGT levels and PSCI using multivariable logistic regression models to estimate odds ratios (ORs) and 95% confidence intervals (CIs) after adjusting for confounding factors. In addition, since GGT is a metabolic index, many factors independent of cognitive status may affect it, notably liver problems. Thus, potential confounders related to liver functions were also taken into account. Restricted cubic spline analyses were performed to assess the association while C statistic, net reclassification improvement (NRI), and integrated discrimination improvement (IDI) were used to evaluate the degree to which the model predicted PSCI after the addition of GGT. We established the conventional model using various parameters, such as age, sex, educational level, BMI, smoking, drinking, NIHSS score at admission, history of stroke, hypertension, dyslipidemia, diabetes mellitus, coronary artery disease, atrial fibrillation, heart failure, and laboratory TC, TG, WBC, as well as UA levels [12, 39–42]. Finally, we performed subgroup analyses, considering age, sex, body mass index, alcohol drinking, stroke type as interaction factors.

All analyses were two-sided, and P<0.05 was considered statistically significant.

## Results

### Baseline characteristics

**Fig. 1:**
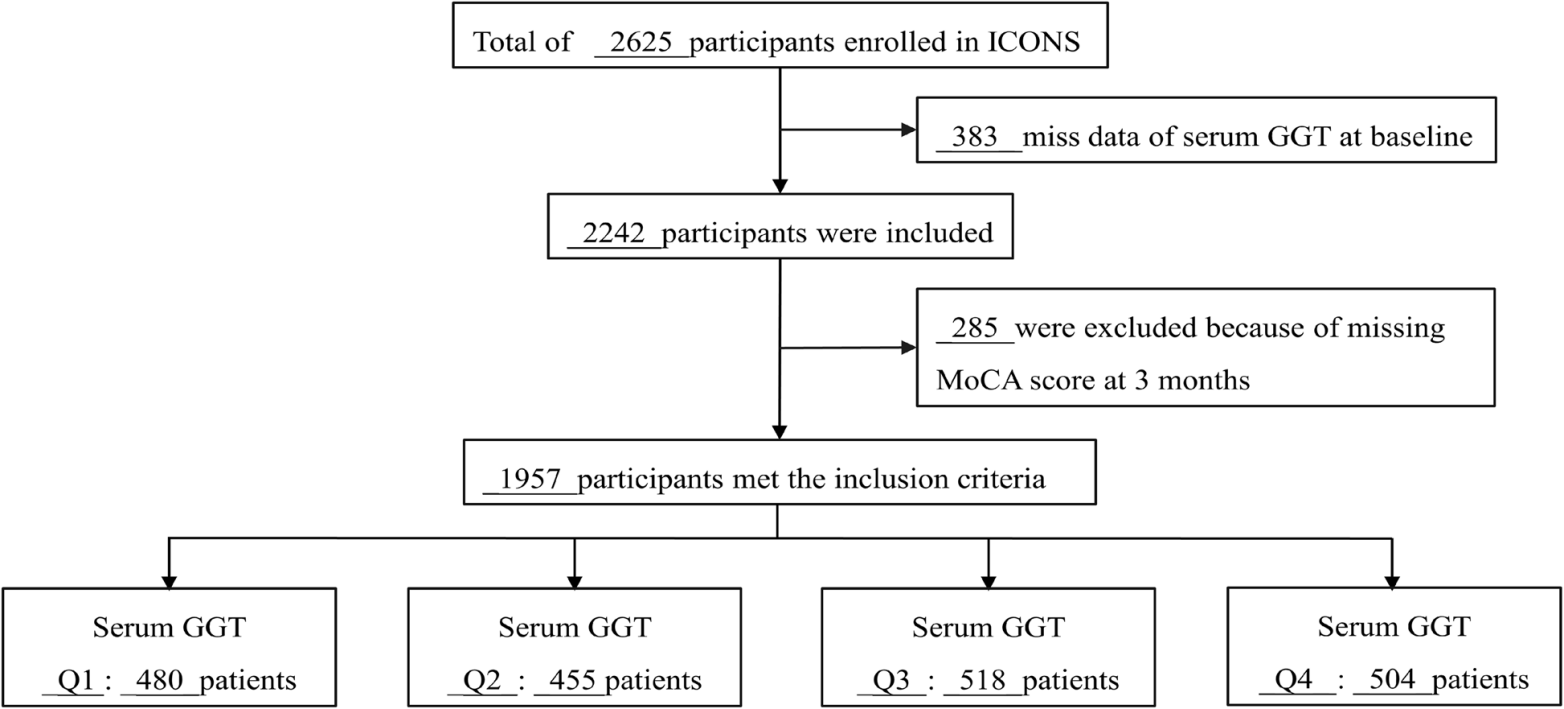
Flowchart in this study. MoCA, The Montreal Cognitive Assessment; GGT, gamma-glutamyl transferase

Among the 2625 participants in the ICONS study, 1957 participants with complete baseline GGT levels and 3-months follow-up were enrolled (Fig. 1). They were divided into four groups according to the quartile of GGT levels, namely < 17, 17 ~ 24, 24 ~ 37, and ≥ 37 U/L. A summary of baseline characteristics of the recruited participants is presented in Tables 1 and 2, Supplementary Table 1. The analysis of these characteristics revealed a significant correlation between GGT levels and age, sex, educational levels, smoking, alcohol drinking, body mass index (BMI), diabetes mellitus, hypoglycemic therapy, antidepressant therapy, sedative-hypnotic therapy, TOAST type, HDL, TG, TC, AST, ALT, UA, eGFR, and albumin.

**Table 1:** Baseline characteristics of the enrolled participants based on their GGT quartiles

Characteristic	Total	GGT level				*P*-value
		Q1(< 17.00)	Q2(17.00-24.00)	Q3(24.00-37.00)	Q4(≥37.00)	
N, (%)	1957	480	455	518	504	
Age, year, median (IQR)	62.00(53.00-69.00)	64.00(58.00-72.00)	63.00(55.00-70.00)	61.00(53.00-68.00)	57.50(52.00-65.00)	< 0.001
Male, n (%)	1419(72.51)	269(56.04)	322(70.77)	393(75.87)	435(86.31)	< 0.001
Education level, n (%)						< 0.001
College or above	198(10.12)	34(7.08)	40(8.79)	63(12.16)	61(12.10)	
High school	453(23.15)	82(17.08)	111(24.40)	139(26.83)	121(24.01)	
Middle school	715(36.54)	179(37.29)	172(37.80)	169(32.63)	195(38.69)	
Elementary or below	509(26.01)	166(34.58)	115(25.27)	128(24.71)	100(19.84)	
Not known	82(4.19)	19(3.96)	17(3.74)	19(3.67)	27(5.36)	
BMI, kg/m^2^, median (IQR)	24.82(23.03-26.85)	24.50(22.29-26.40)	24.57(22.86-26.67)	24.91(23.44-27.06)	25.25(23.53-27.33)	0.003
MoCA scores at admission, median (IQR)	23.00(18.00-26.00)	21.00(16.00-25.00)	23.00(1.008-26.00)	23.00(19.00-26.00)	23.00(19.00-26.00)	< 0.001
Stroke type / Subtype						0.21
AIS	1804(92.18)	435(90.63)	423(92.27)	473(91.31)	473(93.85)	
TIA	153(7.82)	45(9.38)	32(7.03)	45(8.69)	31(6.15)	
Current smoking, n (%)	691(35.31)	132(27.50)	137(30.11)	195(37.64)	227(45.04)	< 0.001
Current drinking, n (%)	357(18.24)	37(7.71)	71(15.60)	95(18.34)	154(30.56)	< 0.001
Medical history, n (%)						
Stroke or TIA	426(21.77)	99(20.63)	110(24.18)	119(22.97)	98(19.44)	0.27
Hypertension	1240(63.36)	291(60.63)	280(61.54)	349(67.37)	320(63.49)	0.12
Diabetes mellitus	447(22.84)	86(17.92)	116(25.49)	135(26.06)	110(21.83)	0.01
Dyslipidemia	202(10.32)	41(8.54)	38(8.35)	61(11.78)	62(12.30)	0.08
Cardiovascular disease	254(12.98)	70(14.58)	58(12.75)	67(12.93)	59(11.71)	0.61
Fatty liver disease	11(0.56)	3(0.63)	1(0.22)	2(0.39)	5(0.99)	0.42
Epilepsy	6(0.31)	2(0.42)	0(0.00)	0(0.00)	4(0.79)	0.05
Cancer	12(0.61)	3(0.63)	0(0.00)	5(0.97)	4(0.79)	0.19
NIHSS on admission, median (IQR)	3.00(1.00-4.00)	3.00(1.00-5.00)	3.00(1.00-4.00)	2.00(1.00-4.00)	2.00(1.00-5.00)	0.68
mRS at admission, median (IQR)	1.00(1.00-2.00)	1.00(1.00-2.00)	1.00(1.00-2.00)	1.00(1.00-2.00)	1.00(1.00-2.00)	0.96
MoCA scores at 3 months, median (IQR)	25.00(21.00-27.00)	23.00(19.00-27.00)	24.00 (21.00-27)	25.00(22.00-28.00)	25.00(22.00-28.00)	< 0.001
Medication use, n (%)						
Antiplatelet aggregation therapy	1913(97.75)	471(98.13)	445(97.80)	508(98.07)	489(97.02)	0.63
Antihypertensive therapy	1240(63.36)	294(61.25)	286(62.86)	323(62.36)	337(66.87)	0.28
Lipid-lowering therapy	1903(97.24)	467(97.29)	438(96.26)	508(98.07)	490(97.22)	0.40
Hypoglycemic therapy	530(27.08)	94(19.58)	135(29.67)	160(30.89)	141(27.98)	0.002
Antidepressant	51(2.61)	18(3.75)	16(3.52)	12(2.32)	5(0.99)	0.03
Sedative-hypnotic	67(3.42)	18(3.75)	24(5.27)	11(2.12)	14(2.78)	0.04
TOAST types, n (%)						0.003
Large-artery atherosclerosis	461(23.56)	112(23.33)	103(22.64)	147(28.38)	99(19.64)	
Cardioembolism	104(5.31)	28(5.83)	19(4.18)	25(4.83)	32(6.35)	
Small-vessel occlusion	491(25.09)	126(26.25)	124(27.25)	115(22.20)	126(25.00)	
Other determined etiology	22(1.12)	12(2.50)	3(0.66)	6(1.16)	1(0.20)	
Undetermined cause	879(44.92)	202(42.08)	206(45.27)	225(43.44)	246(48.81)	
Anxiety state						0.62
None	1593(81.65)	387(80.96)	363(79.96)	432(83.72)	411(81.71)	
Mild	239(12.25)	67(14.02)	62(13.66)	54(10.47)	56(11.13)	
Moderate	63(3.23)	14(2.93)	15(3.30)	17(3.29)	17(3.38)	
Severe	56(2.87)	10(2.09)	14(3.08)	13(2.52)	19(3.78)	

Variables are expressed as median (s) or percentages. Q1, quartile 1 (n - 480): <17 U/L; Q2, quartile 2 (n - 455): 17-24 U/L; Q3, quartile 3 (n - 518): 24-37 U/L; Q4, quartile 4 (n - 504): ≥ 37 U/L. Cardiovascular diseases included atrial fibrillation, coronary heart disease, and heart failure. Medication use included drug use history and treatment during hospitalization. *BMI* Body mass index, *AIS* acute ischemic stroke, *TIA* transient ischemic attack, *NIHSS* the National Institutes of Health Stroke Scale, *mRS* the modified Rankin Scale, *TOAST* the Trial of ORG 10172 in Acute Stroke Treatment, *LDL* low-density lipoprotein, *HDL* high-density lipoprotein, *TG* triglycerides, *TC* total cholesterol, *ALT* alanine aminotransferase, *AST* aspartate aminotransferase, *eGFR* effective glomerular filtration rate, *UA* uric acid, *IQR* interquartile range

**Table 2:** Baseline characteristics of the enrolled participants based on their GGT quartiles

Characteristic	Total	GGT level				*P*-value
		Q1(< 17.00)	Q2(17.00-24.00)	Q3(24.00-37.00)	Q4(≥37.00)	
Laboratory test, median (IQR)						
Serum GGT, U/L	24.00(17.00-37.00)	13.00(11.00-15.00)	19.00(18.00-21.00)	28.20(26.00-31.80)	53.00(42.00-73.00)	< 0.001
LDL, mmol/L	2.41(1.75-3.11)	2.36(1.79-3.03)	2.41(1.73-3.10)	2.43(1.66-3.08)	2.44(1.80-3.21)	0.49
HDL, mmol/L	1.10(0.93-1.31)	1.18(1.00-1.40)	1.10(0.91-1.30)	1.08(0.91-1.26)	1.09(0.90-1.30)	< 0.001
TC, mmol/L	4.10(3.35-4.87)	4.03(3.28-4.80)	4.05(3.34-4.73)	4.15(3.35-4.83)	4.20(3.46-5.11)	0.003
TG, mmol/L	1.38(1.00-1.95)	1.10(0.85-1.54)	1.34(1.00-1.81)	1.45(1.09-2.14)	1.64(1.24-2.29)	< 0.001
ALT, U/L	18.00(13.65-25.90)	14.10(11.90-19.00)	17.00(13.00-22.00)	19.00(14.00-26.00)	25.00(18.00-35.50)	< 0.001
AST, U/L	19.00(16.00-24.00)	17.00(15.00-20.90)	18.00(15.00-23.00)	19.00(16.00-24.00)	21.60(17.00-28.00)	< 0.001
eGFR, ml/min/1.73m^2^	95.11(84.94-103.56)	93.50(84.19-101.72)	94.20(84.43-103.25)	95.45(84.14-103.46)	97.71(88.10-105.75)	< 0.001
UA, μmol/L	294.00(243.00-352.00)	264.00(224.00-322.00)	290.00(242.00-347.00)	298.00(247.00-353.00)	319.00(267.00-379.00)	< 0.001
Albumin, g/L	40.60(38.10-43.00)	39.40(37.30-42.00)	40.50(38.00-42.70)	40.90(38.50-43.20)	41.25(39.00-43.80)	< 0.001

Variables are expressed as median (s) or percentages. Q1, quartile 1 (n - 480): <17 U/L; Q2, quartile 2 (n - 455): 17-24 U/L; Q3, quartile 3 (n - 518): 24-37 U/L; Q4, quartile 4 (n - 504): ≥ 37 U/L. Cardiovascular diseases included atrial fibrillation, coronary heart disease, and heart failure. Medication use included drug use history and treatment during hospitalization. *BMI* body mass index, *AIS* acute ischemic stroke, *TIA* transient ischemic attack, *NIHSS* the National Institutes of Health Stroke Scale, *mRS* the modified Rankin Scale, *TOAST* the Trial of ORG 10172 in Acute Stroke Treatment, *LDL* low-density lipoprotein, *HDL* high-density lipoprotein, *TG* triglycerides, *TC* total cholesterol, *ALT* alanine aminotransferase, *AST* aspartate aminotransferase, *eGFR* effective glomerular filtration rate, *UA* uric acid, *IQR* interquartile range

### Clinical outcomes

**Fig. 2:**
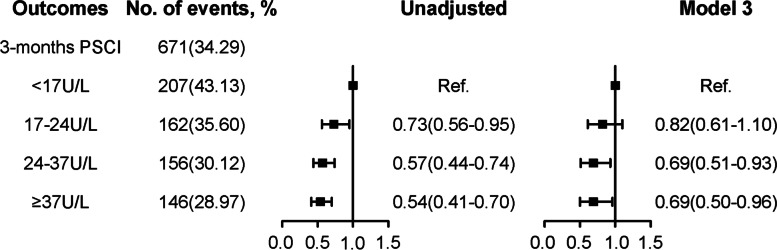
Forest plots of ORs for incident PSCI according to GGT quartile level. The ORs for PSCI incidence according to GGT quartile levels were adjusted for variables of model 3 in Table 3. PSCI, post-stroke cognitive impairment; Ref, reference

Among the eligible participants, 671 (34.29%) patients suffered PSCI at 3 months follow-up, and the characteristics of participants with PSCI were shown in Supplementary Table 2. The correlation between GGT and PSCI is presented in Table 3 and Fig. 2. In Summary, patients in the highest quartile group recorded a 31% decrease in PSCI risk at 3 months follow-up, after adjusting for confounding factors [OR: 0.69 (95%CI: 0.50-0.96)], relative to the lower quartile group.

**Table 3:** Association between GGT levels and PSCI incidence at 3 months follow-up

Outcomes	GGT	No.	Events, N (%)	Unadjusted	Model 1	Model 2	Model 3
				OR (95%CI) *P* value	OR (95%CI) *P* value	OR (95%CI) *P* value	OR (95%CI) *P* value
PSCI	Q1	207	43.13	1	1	1	1
	Q2	162	35.60	0.73(0.56-0.95) 0.02	0.86(0.65-1.13) 0.27	0.83(0.63-1.10) 0.20	0.82(0.61-1.10) 0.18
	Q3	156	30.12	0.57(0.44-0.74) < 0.001	0.73(0.56-0.97) 0.03	0.71(0.53-0.94) 0.02	0.69(0.51-0.93) 0.02
	Q4	146	28.97	0.54(0.41-0.70) < 0.001	0.80(0.60-1.07) 0.14	0.76(0.57-1.02) 0.07	0.69(0.50-0.96) 0.03

Data are presented as OR (95% CI). Set OR of quartile 1 as the reference. Model 1: adjusted by age, sex, educational level; Model 2: adjusted by model 1 plus BMI, medical history, current smoking, current drinking, and medication use (diabetes mellitus, hypoglycemic therapy, antidepressant therapy, sedative-hypnotic therapy); Model 3: adjusted by model 2 plus TOAST type, laboratory tests (LDL, HDL, TC, TG, ALT, AST, eGFR, UA, and Albumin levels); *PSCI* post-stroke cognitive impairment, *GGT* gamma-glutamyl transferase, *BMI* body mass index, *TOAST* the Trial of ORG 10172 in Acute Stroke Treatment, *LDL* low-density lipoprotein, *HDL* high-density lipoprotein; *TG* triglycerides, *TC* total cholesterol, *ALT* alanine aminotransferase, *AST* aspartate aminotransferase, *eGFR* effective glomerular filtration rate, *UA* uric acid, *OR* odds ratio, *CI* confidence interval

**Fig. 3:**
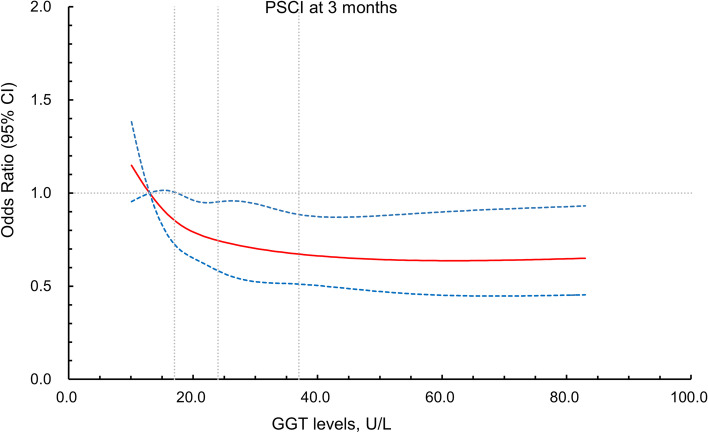
Spline models about the association between GGT levels and clinical outcomes. The association between GGT levels and PSCI occurrence at 3 months. The ORs from the logistic regression model were adjusted for variables of model 3 in Table 3. Red lines indicate adjusted OR, while the blue lines indicate 95%CI. GGT, gamma-glutamyl transferase; PSCI, post-stroke cognitive impairment; OR, odds ratio; CI, confidence interval

Notably, restricted cubic spline analysis revealed that GGT levels were inversely associated with PSCI at 3 months (Fig. 3). However, once GGT increased by over 60 U/L, PSCI incidence no longer decreased.

After incorporating GGT into the conventional model to predict PSCI occurrence, there was a slight improvement in discriminatory power and reclassification after 3 months of follow-up[NRI: 12.00% (*P* = 0.01); IDI: 0.30% (*P* = 0.02)]. Due to the inverse correlation between GGT and PSCI, we set the OR of the highest quartile as the reference (Table 4).

**Table 4:** Reclassification and disclination statistics for PSCI prediction by GGT levels

Clinical outcomes	Model	C-statistic		NRI		IDI	
		Estimate (95% CI)	*P* value	Estimate (95% CI)	*P* value	Estimate (95% CI)	*P* value
PSCI	Conventional model	0.71(0.68-0.73)	0.27	Ref.	0.01	Ref.	0.02
	Conventional model +GGT	0.72(0.69-0.74)		0.12(0.03-0.21)		0.003(0.001-0.01)	

Conventional model: added to factor-adjusted models, including age, sex, educational level, BMI, smoking, drinking, NIHSS score at admission, history of stroke, hypertension, dyslipidemia, diabetes mellitus, coronary artery disease, atrial fibrillation, and heart failure, laboratory test of TC, TG, WBC, UA. TG, triglycerides; *TC* total cholesterol, *WBC* white blood cell count, *UA* uric acid, *GGT* gamma-glutamyl transferase, *PSCI* post-stroke cognitive impairment, *NRI* net reclassification improvement, *IDI* integrated discrimination improvement, *OR* odds ratio, *CI* confidence interval

### Subgroup analysis

**Fig. 4:**
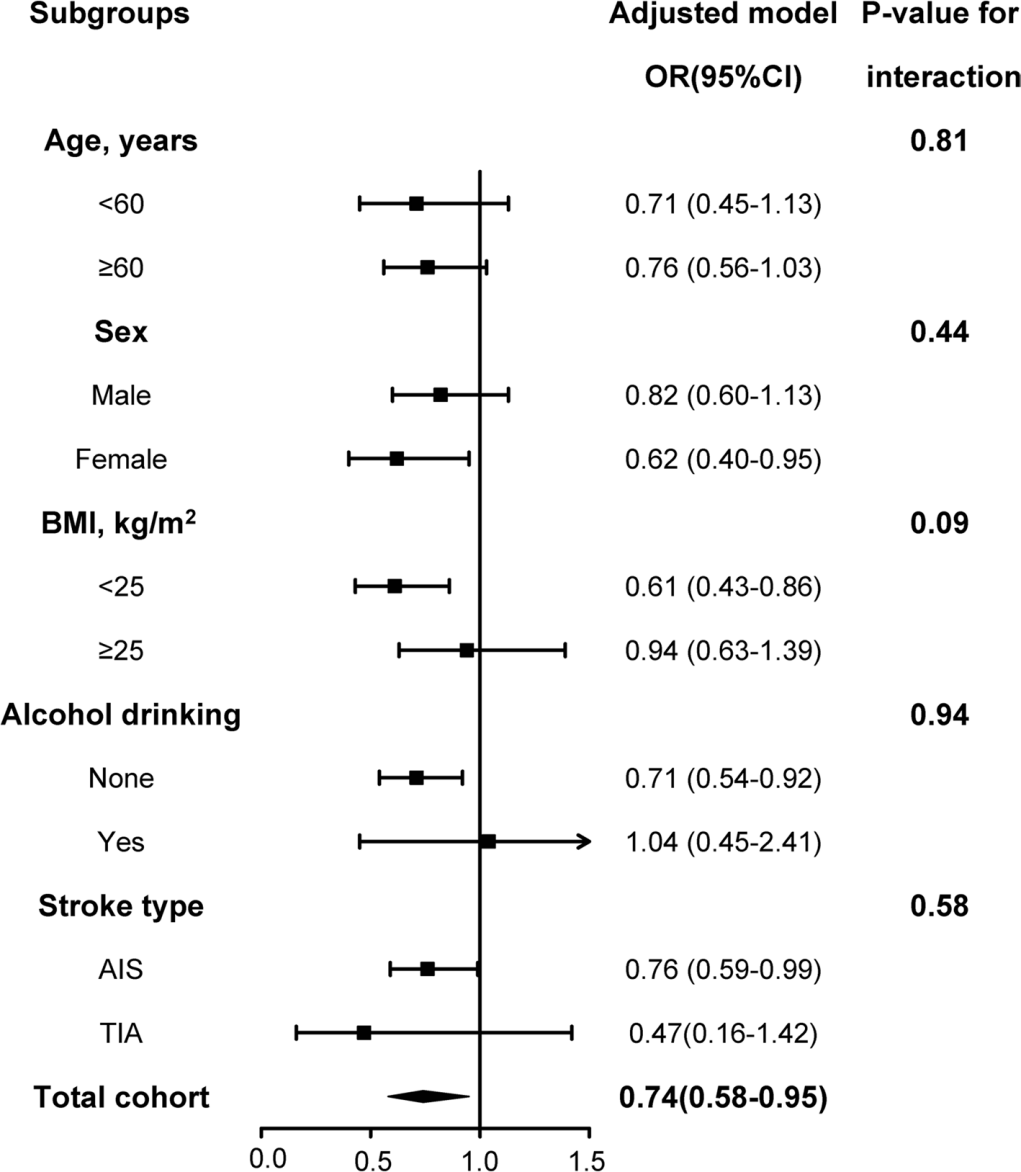
Forest maps of ORs for incident PSCI stratified by different subgroups. Odds ratios for GGT and PSCI were stratified by age, sex, BMI, alcohol drinking, and stroke type. Low-GGT refers to the lowest quartile of 25%, while High-GGT refers to the remaining 75% of the quartile. ORs for incidence of PSCI were adjusted for variables of model 3 in Table 3. GGT, gamma-glutamyl transferase; PSCI, post-stroke cognitive impairment; BMI, body mass index; OR, odds ratio; CI, confidence interval

According to previous studies, some demographic and physiological factors may influence GGT levels, which might result in different effects of GGT on PSCI [24, 28, 47]. Thus, in this study, we further conducted the interaction analysis. Odds ratios for GGT and PSCI were stratified by age, sex, BMI, alcohol drinking, and stroke type. Notably, low- and high-GGT levels refer to the lowest (25%) and highest (75%) quartiles, respectively. *P* values from interaction analyses between GGT and age, sex, BMI, alcohol drinking were 0.91, 0.68, 0.09, and 0.96, stroke type were 0.81, 0.44, 0.09, 0.93, and 0.58, respectively at 3 months of follow-up (Table 5, Fig. 4). This result indicated the underlying interaction effect between BMI and GGT, while other subgroup analyses revealed no significant interactions.

**Table 5:** Subgroup analysis indicating the correlations between GGT levels and PSCI

	Low-GGT No. (%)	High-GGT No. (%)	OR (95%)	*P* value	P _interaction_
Age, years					
<60	46(22.22)	161(77.78)	0.71 (0.45-1.13)	0.15	0.81
≥ 60	161(34.70)	303(65.30)	0.76 (0.56-1.03)	0.08	
Sex					
male	104(23.58)	337(76.42)	0.82(0.60-1.13)	0.23	0.44
female	103(44.78)	127(55.22)	0.62 (0.40-0.95)	0.03	
BMI, kg/m^2^					
<25	127(35.47)	231(64.53)	0.61 (0.43-0.86)	0.004	0.09
≥ 25	80(25.56)	233(74.44)	0.94 (0.63-1.39)	0.75	
Drinking					
None	192(34.47)	365(65.53)	0.71 (0.54-0.92)	0.01	0.93
Yes	15(13.16)	99(86.84)	1.04 (0.45-2.41)	0.92	
Stroke type					
AIS	189 (30.10)	439 (69.90)	0.76 (0.59-0.99)	0.05	0.58
TIA	18 (41.86)	25 (58.14)	0.47(0.16-1.42)	0.18	

Odds ratios for GGT and PSCI were stratified by age, sex, BMI, alcohol drinking, and stroke type. Low-GGT refers to the lowest quartile of 25%, while High-GGT refers to the remaining 75% quartiles. ORs for incidences of PSCI were adjusted for variables of model 3 in Table 3

## Discussion

This large prospective cohort study demonstrated that baseline GGT levels were inversely associated with PSCI occurrence. Specifically, extremely low GGT levels were established to be risk factors for PSCI, even after adjusting for confounding factors including age, sex, educational level, smoking, drinking, BMI, some laboratory indicators, and medical history. Interestingly, the incorporation of GGT into the conventional model resulted in an 11.87% increase in predicting PSCI. Furthermore, PSCI showed a stronger inverse association with GGT especially in individuals with a lower BMI. Correlations between GGT and PSCI in other subgroups revealed no significant change after testing for interactions. This finding indicates that GGT exerted a consistent effect on PSCI, regardless of patients’ age, sex, alcohol drinking habits, and stroke type [48].

As a common complication after stroke, PSCI is associated with serious disabilities. Studies have found that a variety of serum biomarkers are associated with PSCI. Moreover, previous studies reported contrasting findings in terms of the relationship between GGT and cognitive impairment. However, studies have not evaluated the role of GGT in PSCI. According to previous studies, oxidative stress is one of the pathogenic mechanisms of PSCI [30, 49]. After cerebral ischemia and hypoxia, endogenous antioxidants are decreased and oxygen free radicals are overproduced during perfusion of low cerebral blood flow. The body’s oxidative and antioxidant systems are out of balance. Free radicals lead to cell death by damaging proteins, fats, and DNA, which in turn leads to systemic vascular endothelial dysfunction, increases the permeability of the blood-brain barrier and leads to extravasation of blood substances and leakage of serum proteins. These abnormalities are thought to lead to subsequent neuronal damage, such as grey matter atrophy and cortical thinning, leading to cognitive dysfunction [50]. GGT as a biomarker reflecting the oxidation-antioxidant balance in the body should be paid attention to in PSCI related studies. In this multicenter cohort study, we established an association between GGT and PSCI.

This result may be explained by the following mechanism. Biologically, GGT is critical for antioxidant defenses [18]. It is involved in the maintenance of physiological concentrations of glutathione and plays a vital role in protecting cells from oxidative stress damage. GGT induction can be used as a protective adaptation mechanism in physiological and pathological processes [51]. In the initial development of stroke, inflammatory cytokines levels in the body increase, and GGT levels can compensatory increase when catabolism of inflammatory cytokines containing glutathione. In this process, the glutamic acid and the strong reducing agent (dipeptide cysteinyl glycine) are produced. The latter is hydrolyzed by dipeptidase to cysteine and glycine, which are then taken up by cells for intracellular glutathione resynthesis [22, 25]. Among the catabolic products mentioned above, glutamate can be used as the energy material of brain tissue to improve and maintain neurological function. Both glycine and cysteine are constituent amino acids of endogenous antioxidant reduced glutathione, which can protect nerve cells from oxidative stress and reduce the oxidative damage caused by Aβ deposition. The amino acid neurotransmitter is an important transmitter system in the brain. GGT is thought to contribute to the transport process of amino acids across the blood-brain barrier due to the tight junctions of the endothelium cells that prevent the free diffusion of substances [52, 53]. In the brain, GGT mainly exists in the microvascular endothelial cells and in the choroid plexus where the blood-cerebrospinal fluid barrier exists. This enzyme plays a role in regulating the uptake and transport of amino acids, facilitating amino acid transport across the blood-brain barrier and intracellular glutathione regeneration. GGT has been shown to have a protective effect on brain cells. The main deficiency of the vascular endothelial barrier is its inability to prevent the entrance of lipophilic xenobiotic substances, while the intracellular glutathione synthesis catalyzed by GGT can detoxify such substances. Therefore, GGT plays an important role in defending cells from oxidative-induced damage. Moreover, increased GGT levels in the normal range can also indicate that the liver is better at dealing with oxidative stress. When the degree of oxidative stress in the body decreases, PSCI incidence decrease accordingly. Notably, very low GGT levels are indicators of poor liver functions. In patients with more severe chronic liver disease, especially in advanced cirrhosis, the GGT level is continuously at a low value, possibly due to the loss of glutamyl transpeptidase synthesis in hepatocytes. Deficiency or absence of GGT leads to impaired glutamate cycle, which affects the absorption, transport, and utilization of amino acids causes glutathione resynthesis disorder, and progressive neurological symptoms [54]. Therefore, a better GGT reserve is essential for generating enough glutathione to maintain the redox balance in the body.

However, it still had some limitations. First, we adopted a relatively short follow-up period which may have influenced the observed outcomes. Previous studies mainly focused on the relationship between GGT with AD and cognitive decline in later stages of life over 10 year follow-up periods. However, the relationship between GGT and PSCI has not been conclusively determined. Since PSCI is closely associated with stroke and is characterized by its fluctuations, the outcome of the association between GGT and PSCI depends on the length of follow-up. Second, we found that in the population with high GGT levels, levels of biochemical indicators for liver functions, such as ALT and AST were also high. It is possible that this population paid more attention to their health conditions, and on their own, they could be adopting certain measures to protect their liver functions during follow-up. This may have weakened the potentially dangerous relationship between GGT and PSCI. Third, due to missing data for some variables of interest, the potential impact of residual confounders such as a more accurate assessment of the emotional state, partial drug use and dosage, and location of stroke lesions on the results were not excluded completely. Moreover, this study only included patients with minor stroke (low NIHSS score) and TIA, which could not represent all stroke cohorts. To give greater relevance and breadth to the interesting results of this research, further research should also take into account patients with stroke of greater severity and longer follow-up. Furthermore, as an index of biological metabolism, GGT is affected by many factors and presents a dynamic change characteristic. Nevertheless, we only assessed the relationship between GGT at baseline and PSCI. Since baseline GGT levels may be temporarily altered by stroke events, we cannot rule out the possibility of changes in physical health state. The effect of GGT dynamic change on PSCI should be further studied in the future.

## Conclusions

In summary, our results revealed that baseline GGT levels are inversely associated with PSCI, with extremely low GGT levels considered to be a risk factor for PSCI. However, GGT levels dynamically change and it plays a two-sided role in vivo. Therefore, relying solely on GGT to predict PSCI should be carefully considered and further longitudinal studies are needed to clarify the mechanism of GGT affecting neuroplasticity.

## Supplementary Information

**Additional file 1: Supplementary Table 1.** Baseline characteristics of the included and excluded participants. **Supplementary Table 2.** The demographic or neurological differences between PSCI and non-PSCI in included patients.

## Data Availability

The data supporting the findings of this study are available from the corresponding author upon reasonable request.

## Abbreviations

GGT: Gamma-glutamyl transferase
PSCI: Post-stroke cognitive impairment
VCI-ND: Vascular cognitive impairment-no dementia
MoCA: The Montreal Cognitive Assessment
BMI: Body mass index
AIS: Acute ischemic stroke
TIA: Transient ischemic attack
TOAST: Trial of ORG 10172 in Acute Stroke Treatment
HDL: High-density lipoprotein
LDL: Low-density lipoprotein
TG: Triglycerides
TC: Total cholesterol
ALT: Alanine aminotransferase
AST: Aspartate aminotransferase
eGFR: Effective glomerular filtration rate
UA: Uric acid
IQR: Interquartile range
OR: Odds ratios
CI: Confidence interval
NRI: Net reclassification improvement
IDI: Integrated discrimination improvement
ICONS: The Impairment of Cognition and Sleep study
CNSR-3: The China National Stroke Registry-3
