# Supplementary material for: Gamma-glutamyl transferase levels are associated with the occurrence of post-stroke cognitive impairment: a multicenter cohort study
Source: BMC Neurol. 2022 Feb 23;22:65. doi: 10.1186/s12883-022-02587-4 (PMC8864864; doi:10.1186/s12883-022-02587-4)
Supplement: Supplementary file 1 — Additional file 1: Supplementary Table 1. Baseline characteristics of the included and excluded participants. Supplementary Table 2. The demographic or neurological differences between PSCI and non-PSCI in included patients. [file 12883_2022_2587_MOESM1_ESM.docx]

Supplementary Table 1 Baseline characteristics of the included and excluded participants

| Characteristic | Total | Included patients | Excluded patients | P-value |
| --- | --- | --- | --- | --- |
| N, (%) | 2625 | 1957 | 668 |  |
| Age, year, median (IQR) | 61.00(53.00-68.00) | 62.00(53.00-69.00) | 61.00(53.00-67.00) | 0.12 |
| Male, n (%) | 1900(72.38) | 1419(72.51) | 481(72.01) | 0.80 |
| Education level, n (%) |  |  |  | 0.14 |
| College or above | 262(9.98) | 198(10.12) | 64(9.58) |  |
| High school | 625(23.81) | 453(23.15) | 172(25.75) |  |
| Middle school | 940(35.81) | 715(36.54) | 225(33.68) |  |
| Elementary or below | 675(25.71) | 509(26.01) | 166(24.85) |  |
| Not known | 123(4.69) | 82(4.19) | 41(6.14) |  |
| BMI, kg/m^2^, median (IQR) | 24.82(22.99-26.89) | 24.82(23.03-26.85) | 24.80(22.86-27.04) | 0.89 |
| Current smoking, n (%) | 941(35.85) | 691(35.31) | 250(37.43) | 0.32 |
| Current drinking, n (%) | 502(19.12) | 357(18.24) | 145(21.71) | 0.05 |
| Stroke type / Subtype |  |  |  | 0.12 |
| AIS | 2432(92.65) | 1804(92.18) | 628(94.01) |  |
| TIA | 193(7.35) | 153(7.82) | 40(5.99) |  |
| Medical history, n (%) |  |  |  |  |
| AIS or TIA | 581(22.13) | 426(21.77) | 155(23.20) | 0.44 |
| Hypertension | 1642(62.55) | 1240(63.36) | 402(60.18) | 0.14 |
| Diabetes mellitus | 599(22.82) | 447(22.84) | 152(22.75) | 0.96 |
| Dyslipidemia | 259(9.87) | 202(10.32) | 57(8.53) | 0.18 |
| Cardiovascular disease | 347(13.22) | 254(12.98) | 93(13.92) | 0.53 |
| Fatty liver disease | 13(0.50) | 11(0.56) | 2(0.30) | 0.54 |
| Epilepsy | 7(0.27) | 6(0.31) | 1(0.15) | 0.69 |
| Cancer | 18(0.69) | 12(0.61) | 6(0.90) | 0.44 |
| NIHSS on admission, median (IQR) | 3.00(1.00-5.00) | 3.00(1.00-4.00) | 3.00(1.00-6.00) | <0.001 |
| mRS at admission, median (IQR) | 1.00(1.00-2.00) | 1.00(1.00-2.00) | 1.00(1.00-3.00) | <0.001 |
| Medication use, n (%) |  |  |  |  |
| Antiplatelet aggregation therapy | 2569(97.87) | 1913(97.75) | 656(98.20) | 0.49 |
| Antihypertensive therapy | 1639(62.44) | 1240(63.36) | 399 (59.73) | 0.09 |
| Lipid-lowering therapy | 2551 (97.18) | 1903 (97.24) | 648(97.01) | 0.75 |
| Hypoglycemic therapy | 726(27.66) | 530(27.08) | 196(29.34) | 0.26 |
| Antidepressant | 63(2.40) | 51(2.61) | 12(1.80) | 0.24 |
| Sedative-hypnotic | 85(3.24) | 67(3.42) | 18(2.69) | 0.36 |
| TOAST types, n (%) |  |  |  | 0.31 |
| Large-artery atherosclerosis | 631(24.04) | 461(23.56) | 170(25.45) |  |
| Cardioembolism | 147(5.60) | 104(5.31) | 43(6.44) |  |
| Small-vessel occlusion | 657(25.03) | 491(25.09) | 166(24.85) |  |
| Other determined etiology | 25(0.95) | 22(1.12) | 3(0.45) |  |
| Undetermined cause | 1165(44.38) | 879(44.92) | 286(42.81) |  |
| A[nxiety](javascript:;) [state](javascript:;) |  |  |  | 0.04 |
| None | 2137(81.75) | 1593(81.65) | 544(82.05) |  |
| Mild | 334(12.78) | 239(12.25) | 95(14.33) |  |
| Moderate | 79(3.02) | 63(3.23) | 16(2.41) |  |
| Severe | 64(2.45) | 56(2.87) | 8(1.21) |  |
| Laboratory test, median (IQR) |  |  |  |  |
| Serum GGT, U/L | 24.00(17.00-38.00) | 24.00(17.00-37.00) | 25.10(18.00-39.00) | 0.08 |
| LDL, mmol/L | 2.41(1.77-3.09) | 2.41(1.75-3.11) | 2.41(1.81-3.08) | 0.50 |
| HDL, mmol/L | 1.10(0.92-1.30) | 1.10(0.93-1.31) | 1.09(0.90-1.29) | 0.03 |
| TC, mmol/L | 4.11(3.38-4.85) | 4.10(3.35-4.87) | 4.12(3.47-4.78) | 0.40 |
| TG, mmol/L | 1.37(1.01-1.93) | 1.38(1.00-1.95) | 1.32(1.02-1.86) | 0.33 |
| ALT, U/L | 18.00(13.00-25.50) | 18.00(13.65-25.90) | 17.00(12.70-25.00) | 0.02 |
| AST, U/L | 19.00(16.00-24.00) | 19.00(16.00-24.00) | 19.00(15.95-23.45) | 0.88 |
| eGFR, ml/min/1.73m2 | 95.26(84.88-103.76) | 95.11(84.94-103.56) | 95.86(84.56-103.92) | 0.81 |
| UA, μmol/L | 296.00(244.00-355.00) | 294.00(243.00-352.00) | 300.00(251.00-365.00) | 0.03 |
| Albumin, g/L | 40.50(38.10-43.00) | 40.60(38.10-43.00) | 40.30(38.00-43.00) | 0.21 |

Variables are expressed as median (s) or percentages. Q1, quartile 1 (n - 480): ˂17 U/L; Q2, quartile 2 (n - 455): 17-24 U/L; Q3, quartile 3 (n - 518): 24-37 U/L; Q4, quartile 4 (n - 504): ≥ 37 U/L. Cardiovascular diseases included atrial fibrillation, coronary heart disease, and heart failure. Medication use included drug use history and treatment during hospitalization. BMI, body mass index; AIS, acute ischemic stroke; TIA, transient ischemic attack; NIHSS, the National Institutes of Health Stroke Scale; mRS, the modified Rankin Scale; TOAST, the Trial of ORG 10172 in Acute Stroke Treatment; LDL, low-density lipoprotein; HDL, high-density lipoprotein; TG, triglycerides; TC, total cholesterol; ALT, alanine aminotransferase; AST, aspartate aminotransferase; eGFR, effective glomerular filtration rate; UA, uric acid; IQR, interquartile range.

Supplementary Table 2 The demographic or neurological differences between PSCI and non-PSCI in included patients

| Characteristic | PSCI patients | non-PSCI patients | P-value |
| --- | --- | --- | --- |
| N, (%) | 671 | 1286 |  |
| Age, year, median (IQR) | 65.00(57.00-72.00) | 60.00(52.00-67.00) | <0.001 |
| Male, n (%) | 441(65.72) | 978(76.05) | <0.001 |
| Education level, n (%) |  |  | <0.001 |
| College or above | 30(4.47) | 168(13.06) |  |
| High school | 113(16.84) | 340(26.44) |  |
| Middle school | 233(34.72) | 482(37.48) |  |
| Elementary or below | 239(18.58) | 270(40.24) |  |
| Not known | 25(3.73) | 57(4.43) |  |
| BMI, kg/m^2^, median (IQR) | 24.80(22.86-27.01) | 24.83(23.06-26.81) | 0.64 |
| MoCA scores at admission, median (IQR) | 18.00(13.00-21.00) | 24.00(22.00-27.00) | <0.001 |
| Current smoking, n (%) | 220(32.79) | 471(36.63) | 0.09 |
| Current drinking, n (%) | 114(16.99) | 243(18.90) | 0.30 |
| Stroke type / Subtype |  |  | 0.10 |
| AIS | 628(93.59) | 1176(91.45) |  |
| TIA | 43(6.41) | 110(8.55) |  |
| Medical history, n (%) |  |  |  |
| AIS or TIA | 192(28.61) | 234(18.20) | <0.001 |
| Hypertension | 446(66.47) | 794(61.74) | 0.04 |
| Diabetes mellitus | 168(25.04) | 279(21.70) | 0.09 |
| Dyslipidemia | 56(8.35) | 146(11.35) | 0.04 |
| Cardiovascular disease | 91(13.56) | 163(12.67) | 0.58 |
| Fatty liver disease | 1(0.15) | 10(0.78) | 0.08 |
| Epilepsy | 1(0.15) | 5(0.39) | 0.36 |
| Cancer | 4(0.60) | 8(0.62) | 0.94 |
| NIHSS on admission, median (IQR) | 3.00(1.00-5.00) | 2.00(1.00-4.00) | 0.01 |
| mRS at admission, median (IQR) | 2.00(1.00-3.00) | 1.00(1.00-2.00) | <0.001 |
| MoCA scores at 3 months, median (IQR) | 19.00(16.00-21.00) | 26.00(25.00-28.00) | <0.001 |
| Medication use, n (%) |  |  |  |
| Antiplatelet aggregation therapy | 651(97.02) | 1262(98.13) | 0.11 |
| Antihypertensive therapy | 447(66.62) | 793(61.66) | 0.03 |
| Lipid-lowering therapy | 657(97.71) | 1246(96.89) | 0.19 |
| Hypoglycemic therapy | 195(29.06) | 335(26.05) | 0.15 |
| Antidepressant | 19(2.83) | 32(2.49) | 0.65 |
| Sedative-hypnotic | 36(5.37) | 31(2.41) | <0.001 |
| TOAST types, n (%) |  |  | <0.001 |
| Large-artery atherosclerosis | 196(29.21) | 265(20.61) |  |
| Cardioembolism | 42(6.26) | 62(4.82) |  |
| Small-vessel occlusion | 148(22.06) | 343(26.67) |  |
| Other determined etiology | 10(1.49) | 12(0.93) |  |
| Undetermined cause | 275(40.98) | 604(46.97) |  |
| A[nxiety](javascript:;) [state](javascript:;) |  |  | 0.002 |
| None | 524(78.33) | 1069(93.39) |  |
| Mild | 85(12.71) | 154(12.01) |  |
| Moderate | 31(4.63) | 32(2.50) |  |
| Severe | 29(4.33) | 27(2.11) |  |
| Laboratory test, median (IQR) |  |  |  |
| Serum GGT, U/L | 21.10(15.00-33.00) | 25.00(17.50-39.00) | <0.001 |
| LDL, mmol/L | 2.53(1.86-3.22) | 2.36(1.71-3.03) | <0.001 |
| HDL, mmol/L | 1.12(0.94-5.05) | 1.10(0.92-1.30) | 0.004 |
| TC, mmol/L | 4.24(3.45-5.05) | 4.04(3.31-4.80) | <0.001 |
| TG, mmol/L | 1.34(0.98-1.86) | 1.41(1.02-1.99) | 0.03 |
| ALT, U/L | 18.00(13.00-24.10) | 19.00(14.00-26.00) | 0.11 |
| AST, U/L | 19.00(15.90-24.00) | 19.00(16.00-24.00) | 0.40 |
| eGFR, ml/min/1.73m2 | 93.42(84.04-101.83) | 95.85(85.58-104.75) | 0.003 |
| UA, μmol/L | 286.00(236.00-348.00) | 297.90(247.00-354.00) | 0.01 |
| Albumin, g/L | 40.30(38.00-42.60) | 40.75(38.20-43.10) | 0.04 |

Variables are expressed as median (s) or percentages. Q1, quartile 1 (n - 480): ˂17 U/L; Q2, quartile 2 (n - 455): 17-24 U/L; Q3, quartile 3 (n - 518): 24-37 U/L; Q4, quartile 4 (n - 504): ≥ 37 U/L. Cardiovascular diseases included atrial fibrillation, coronary heart disease, and heart failure. Medication use included drug use history and treatment during hospitalization. BMI, body mass index; AIS, acute ischemic stroke; TIA, transient ischemic attack; NIHSS, the National Institutes of Health Stroke Scale; mRS, the modified Rankin Scale; TOAST, the Trial of ORG 10172 in Acute Stroke Treatment; LDL, low-density lipoprotein; HDL, high-density lipoprotein; TG, triglycerides; TC, total cholesterol; ALT, alanine aminotransferase; AST, aspartate aminotransferase; eGFR, effective glomerular filtration rate; UA, uric acid; IQR, interquartile range.
